# Exploring the Role of Transcriptomics, Proteomics, and Machine Learning in HPV Infection and Cardiovascular Disease

**DOI:** 10.3390/biomedicines13122942

**Published:** 2025-11-29

**Authors:** Lisa Lazzari, Ilaria Casati, Sarah Wang, Melanie J. Hezzell, Gianni D. Angelini, Tim Dong

**Affiliations:** 1 School of Medicine and Surgery, Università degli Studi di Milano-Bicocca, 20126 Milano, Italy; 2 Severn Pathology Cellular Pathology, Southmead Hospital, North Bristol NHS Trust, Bristol BS10 5NB, UK; 3 Bristol Veterinary School, University of Bristol, Langford House, Langford, Bristol BS40 5DU, UK; 4 Bristol Heart Institute, Translational Health Sciences, University of Bristol, Bristol BS2 8HW, UK

**Keywords:** bioinformatics, transcriptomics, proteomics, cardiovascular disease, infectious disease, machine learning, multimorbidity

## Abstract

Background: Human papillomavirus (HPV) is a serious disease caused by a viral infection that can lead to various types of cancers in both women and men. Nearly all cases of cervical cancer (99.7%) develop as a result of an HPV infection, ranging from low to high grade, with a 5-year mortality rate ranging from 8 to 81% depending on the timeliness of diagnosis. Recent studies have further shown that HPV significantly increases the risk of cardiovascular disease, including coronary artery disease (CAD). However, the mechanism and impact of HPV on CVD from a proteomics and transcriptomics perspective are not well understood. Objectives: The purpose of this work is to provide the evidence framework for using machine learning to further advance knowledge on the interplay of HPV and CVD in relation to proteomic and transcriptomic changes. Key findings: In addition to existing known relationships between HPV and atherosclerosis and CAD, dilated cardiomyopathy (DCM) is identified as an important cardiovascular disease modified by HPV infections. A more comprehensive understanding of the cholesterol-modifying mechanisms underpinning HPV’s influence on CVD has been identified. Downstream ML has been used to selectively identify key proteins for subsequent bioinformatic mining across a range of public and in-house curated databases. Implications: By further understanding the mechanisms underlying HPV-induced cardiovascular pathogenesis, machine learning models can be developed in a more targeted manner, stratifying patients that will have an optimal response to emerging probiotic-based therapies.

## 1. Introduction

### 1.1. Overview of HPV Infection and Global Burden

Human papillomavirus (HPV) is a serious disease caused by a viral infection that can lead to various types of cancers in both women and men. HPV comprises more than 200 different strains that cause either benign or malignant lesions by infecting the basal cells of squamous stratified epithelia [1]. High-risk HPV strains (HR-HPV) are linked to the development of cervical carcinoma because they can integrate their HPV DNA into the host epithelial cells’ genome, triggering the expression of viral genes that produce the oncoproteins *E6* and *E7*. By disrupting normal cell regulation, these proteins may facilitate malignant transformation and progression to invasive carcinoma [2].

Globally, the prevalence of all types of HPV combined is estimated to be 31%, with high-risk strains accounting for 21% of infections [3]. Among these, HPV-16 is the most common genotype, with a 5% prevalence rate, followed by HPV-6, with a 4% prevalence rate [3]. Nearly all cases of cervical cancer (99.7%) develop as a result of an HPV infection [4], with prognosis strongly dependent on the stage at diagnosis, as reflected by the 5-year mortality rate ranging from 8 to 81% [5]. Cervical cancer ranks nineth in terms of global disease occurrence, with more than 600,000 new cases reported since 2020, and fourth when considering exclusively the female population [6]. In Europe alone, an estimated 58,219 new cases and 26,950 deaths occurred in 2022 [7].

However, HPV is a disease affecting not only women but also men, with approximately 1 in 3 men aged 15 or older infected with at least one strain of HPV, and 1 in 5 with at least one of the high-risk HPV strains [8]. Evidence shows that HPV causes anal cancer, 30–50% of penile cancers [9], oral cancers, and an increasing proportion of oropharyngeal cancers (incidence rate of 140,000 per year [10]) in men [1]. The virus more commonly affects certain groups, including women living with HIV, men who have sex with men, immunocompromised individuals, people who are co-infected with other sexually transmitted infections (STIs), those undergoing immunosuppressive treatment, and children who have been sexually abused [11].

### 1.2. Emerging Evidence Linking HPV to Cardiovascular Disease (CVD)

Beyond its well-established oncogenic effects, HPV has increasingly been implicated in cardiovascular disease (CVD) [12]. CVDs are the leading causes of mortality worldwide, accounting for an estimated 17.9 million fatalities (32% of all global deaths) in 2019 and anticipated to reach 23.6 million by 2030 [8]. Conventional risk factors are insufficient to explain the elevated incidence and prevalence of CVD [13], with approximately 20% of cases arising in the absence of known risk factors (i.e., idiopathic) [8]. Recent studies have shown that HPV, particularly high-risk genotypes such as HPV-16 and HPV-18, significantly increases the risk of CVD, including coronary artery disease (CAD), especially among menopausal and obese women [13]. In one cohort of 9,353 women aged 20–59, 40.8% were found positive for HPV DNA, and 3.0% subsequently developed CVD; vaginal HPV infection was found to be significantly associated with CVD (OR = 1.54, 95% CI 1.15–2.08), and this association was not observed in the patients vaccinated against HPV [14]. Another investigation using the National Health and Nutrition Examination Survey (NHANES) dataset reported that HPV-positive women had a higher risk of CVD, with those in the climacteric phase who were HPV positive having a threefold higher risk of developing CAD than those without HPV infection [14]. Supporting these findings, polymerase chain reaction (PCR) testing detected HPV DNA in 50% of patients with coronary artery atherosclerosis.

The precise biological mechanisms explaining these associations are still being elucidated. HPV is believed to influence the development of CAD through damage to the vascular endothelium and the induction of systemic inflammation [8]. Parallel to this effect, HPV disturbs host lipid metabolism, promoting the development or worsening of atherosclerosis. These insights are supported by a large 17-year prospective study of 163,250 Korean women, showing elevated CVD mortality in those with high-risk HPV infection, particularly when obesity was present [13].

### 1.3. Need for Multi-Omics and Machine Learning Integration to Uncover Mechanisms

Although epidemiological evidence increasingly supports a link between HPV infection and CVD, the underlying biological mechanisms remain poorly defined. Current proteomic and transcriptomic data are limited. In addition, a holistic view of the application of machine learning approaches related to this area and whether the incorporation of proteomics for such modelling would be beneficial is not clear. Therefore, this review aims to evaluate the role of machine learning approaches in advancing our understanding of the interplay between HPV infection and cardiovascular disease, with particular emphasis on proteomic and transcriptomic insights.

Longitudinal studies will be required to further ascertain the long-term impact of HPV infection on CVD, including CAD, with appropriate adjustment for various confounders such as obesity and smoking [8]. In addition, research involving larger populations and experimental models is necessary to better understand the mechanisms by which HPV contributes to atherogenesis in both men and women.

The subsequent section aims to further explore potential mechanistic effects of HPV infection on heart disease by bridging the gap between these two disparate conditions.

## 2. Relationship Between HPV Biomarkers and Cardiovascular Disease

In order to reconcile conflicting or incomplete data regarding specific molecular pathways in cardiovascular pathology, we meticulously checked the Supplementary Materials (e.g., differential expressions, network interaction, enrichment profiles) of each publication and cross-checked across multiple databases such as UniProt, GeneCards, and STRING, as well as across the literature, combining our internal expertise to ensure the mechanisms were scientifically plausible.

Table 1 presents several proteomic markers (human and viral) for HPV pathogenesis, categorised by different tissues. Whilst the genetics of HPV are frequently studied and well understood, the proteomic changes induced by HPV infection are less well understood. For this reason, as well as practical constraints (e.g., resources, time) in relation to this project, genomic markers were beyond the scope of the current study and were not considered in any detail here. Nonetheless, transcriptomics markers were included since transcriptomics offer high resolution with a large dynamic range, providing time-sensitive datasets (since the transcriptome is expected to vary more across time than the proteome) that are available in high volume [15]. The markers identified by both proteomics and transcriptomics methods are in bold.

### 2.1. Transcriptomics

#### 2.1.1. E6, E7 Genes

***E6* and *E7* genes** in HR-HPVs are transcribed in a single pre-mRNA. Analyses of alternative splicing isoforms showed that E6*I was the most abundant transcript in HPV16 and HPV18, resulting from a splicing event that allowed *E7* translation [27]. *E7* protein binds to a region of the retinoblastoma tumour suppressor protein (Rb) essential for its tumour-suppressor function; it disrupts the interaction between Rb and E2F (a family of transcription factors), releasing E2F factors in their transcriptionally active forms. This stimulates replication and cell division, disrupting the normal physiological functions of the specific tumour suppressor gene [42]. Regarding their potential association with cardiovascular diseases, HPV16 *E6* and *E7* oncoproteins have been linked to increased COX-2 expression, further linking HPV oncogenes to potent inflammatory cascades that intensify the progression of atherosclerosis. *E6* and *E7* oncoproteins also dysregulate cellular processes involved in WNT pathway activation [8].

#### 2.1.2. CCNB2 (Cyclin B2)

**CCNB2** acts as a cell cycle checkpoint during the G2/M transition. Its aberrant expression frequently contributes to tumorigenesis [43]. Studies have shown that CCNB2 is overexpressed in various HPV-positive cancers, such as cervical squamous cell carcinoma, and is being investigated as a potential biomarker [29].

#### 2.1.3. Id1 (DNA-Binding Protein Inhibitor ID-1)

***Id genes*** regulate the proliferation and differentiation of embryonic stem cells by controlling cell cycle phase transitions. Among them, Id1, as a helix-loop-helix (HLH) transcription factor, promotes proliferation, inhibits differentiation, and mediates senescence by negatively regulating bHLH (basic HLH) transcription factors, such as c-Myc and Hif1α, that are central regulators of aerobic glycolysis and glutaminolysis. *Id-1 RNA* and protein expression are significantly increased in transformed cervical epithelial cells, suggesting a possible role of Id-1 in their transformation [31]. Multiple studies indicate that Id1 is regulated by shear stress and may act as an important mechano-responsive factor. Future research should explore whether shear stress–induced changes in Id1 alter the functional state of endothelial cells (ECs) and contribute to plaque inflammation and instability [44].

#### 2.1.4. MicroRNAs: miR-146a-5p, miR-9-5p, and miR-363-3p

**MicroRNAs** (miRNAs) are a class of non-coding RNA that regulate gene expression post-transcriptionally, thereby influencing a wide range of cellular processes. Different miRNAs have been labelled as tumour-promoting (oncogenic miR or oncomiR) or tumour suppressive based on the nature of their target genes. OncomiRs can repress tumour suppressor genes expression and are frequently upregulated in the diseased state. In HPV-associated cancers, several miRNAs have been identified as differentially expressed, and many of these same molecules also play important roles in cardiovascular disease (CVD).

**miR-146a-5p.** One example is miR-146a-5p, which has been consistently upregulated in cervical cancers, and transfecting it leads to a significant increase in cell proliferation [45]. Beyond its role in oncogenesis, miR-146a-5p is also implicated in cardiovascular pathology. It is released from the heart after ischemic injury and acts as a potent pro-inflammatory mediator. Moreover, it also activates cardiomyocytes, cardiac fibroblasts, and resident macrophages through TLR7 signalling, inducing cytokine production and chemokine release (TNF-α, IL-6, and IL-1b), leading to endothelial barrier dysfunction, immune cell infiltration, and reduced cardiomyocyte contractility. These processes collectively contribute to plaque instability and the progression of cardiovascular disease [46]. Additionally, in a recent UK BioBank Pharma Proteomics Project (PPP) study, it was found that participants with coronary artery disease during follow-up were enriched for apoptosis-related proteins such as those from the tumour necrosis factor (TNF) receptor family [47].

Another important candidate is ***miR-9-5p***, identified in profiling studies comparing HPV-positive and HPV-negative cancers. It was markedly upregulated in HPV-positive tumours, whereas it was absent in HPV-negative specimens. This observation is in line with the broader oncogenic associations of *miR-9-5p* across several human cancers [36,48]. In the cardiovascular system, miR-9-5p mediates hypoxic injury in cardiomyoblasts by suppressing protective signalling pathways (PPARδ, MDM2) and promoting mitochondrial dysfunction, leading to excessive reactive oxygen species (ROS) accumulation; its suppression has been shown to prevent adverse remodelling following acute myocardial infarction [49]. It also promotes fibrosis through the Smad7/Smad3 pathway, underlining its contribution to both structural and functional cardiac pathology [50].

A similar pattern is seen with ***miR-363-3p***, which has also been found to be upregulated in HPV-positive tumour samples [37]. In the context of CVD, elevated circulating levels of *miR-363-3p* have been detected in patients with acute myocardial infarction, where it has been proposed as a potential diagnostic biomarker. Functionally, overexpression of this miRNA exacerbates endothelial injury, while its downregulation improves outcomes in models of myocardial infarction [51].

High expression levels of TMEM45A and p16INK4a have been linked to an increased risk of high-grade neoplasia in women infected with HPV16 [35].

#### 2.1.5. TMEM45A (Transmembrane Protein 45A)

**Transmembrane protein 45A (TMEM45A)** is a transmembrane protein whose precise function remains unclear, although several studies have shown that TMEM45A is a hypoxia-inducible gene [52] with anti-apoptotic properties under genotoxic stress. It is highly expressed in human keratinocytes [53] and has been associated with keratinocyte differentiation. These features support the notion that early overexpression of TMEM45A may serve as a marker for progression to high-grade cervical lesions in women carrying high-risk HPV. In relation to CV disease, TMEM45A has been upregulated in atrial tissue from patients with atrial fibrillation (AF) [54].

#### 2.1.6. p16INK4a

The **p16INK4a** protein is a cyclin-dependent kinase (CDK) inhibitor that becomes strongly upregulated in response to cellular stress, including the presence of HPV *E7* oncoprotein. Accordingly, HPV-associated tumours display high levels of p16INK4a.

p16INK4a is also recognised as a contributor to CV disease. As a regulator of the cell cycle, its overexpression is linked with processes of cellular senescence that can impair tissue repair and promote age-related pathology. p16INK4a overexpression has been implicated in vascular calcification [55] and in limiting the proliferative and migratory capacity of cardiac cells after myocardial infarction—thereby hindering cardiac repair after injury [56].

#### 2.1.7. K17 (Stress Keratin 17)

**Stress keratin 17 (K17)** is a stress-induced keratin expressed in epithelial cells under conditions such as wound healing, inflammation, and autoimmune diseases. It is overexpressed in multiple cancer types, including HPV+ head and neck SCC [39].

It also plays a role in CV disease, particularly in the setting of inflammation and plaque instability. Because its expression is elevated under conditions of stress, K17 may contribute to both the development and progression of cardiovascular disease. It is also thought to influence the immune response within blood vessels, promoting inflammation and destabilising atherosclerotic plaques [57].

### 2.2. Proteomics

#### 2.2.1. p16

**p16** is a cyclin-dependent kinase inhibitor that negatively affects cell proliferation by regulating the G1-S transition of the cell cycle. It exerts its effect by binding to CDK4 and CDK6, preventing phosphorylation of the retinoblastoma protein (pRb). This results in the blockade of E2F-mediated transcription of DNA synthesis genes. The p16 protein is upregulated in high-risk HPV infections, since the viral protein E7 inactivates pRb, leading to compensatory p16 overexpression [58,59]. For this, p16 has been suggested as a surrogate marker for HPV infection due to its differential expression across HPV+ and HPV− samples [23]. In relation to the heart, p16 has been shown to play a key role in cardiomyocyte senescence, whereby higher levels of expression are associated with ageing, myocardial infarction, and cardiac remodelling and dysfunction.

#### 2.2.2. TP73 (Tumour Protein p73)

**Tumour protein p73** (**TP73**) is a protein that encourages apoptosis in cells in response to DNA damage. TP73 has been shown to have significantly higher expression in HPV+ tumour cells than HPV− tumour cells [23]. This protein has multiple isoforms, which may be either anti-oncogenic (e.g., TAp73) or oncogenic (e.g., DNp73). Indeed, TAp73 mainly plays anti-transformative roles (e.g., blocking epithelial-to-mesenchymal transition), while DNp73 performs an anti-apoptotic function, presumably by interfering with p53 and/or TAp73, thereby favouring cell proliferation [60]. Expression levels of this protein are generally low in the heart, though the specific isoform DNp73 has been found to activate the proliferation of cardiomyocytes by inhibiting the tumour suppressor protein p53 [61].

#### 2.2.3. HP (Haptoglobin)

**Haptoglobin (HP)** is a protein that helps to counter the oxidative-damaging effects of free haemoglobin as a result of haemolysis or bleeding by capturing it for macrophage removal. It is also associated with additional antioxidant and anti-microbial properties. One of its isoforms, glycoform 9, was found to be enriched in HPV+ tumours compared to HPV− tumours [10]. Increased post-translational modifications of this protein have also been observed in other cancers (e.g., lung and ovarian) [10]. One of its three isoforms, Hp2-2, is associated with a 2.4-fold higher risk of ischaemic cardiovascular mortality [62]. The negative effects of this isoform are at least partly due to its disruption of the reverse cholesterol transport process and of cholesterol balance in the body, as it strongly binds human apolipoprotein A-I and apolipoprotein E [62].

#### 2.2.4. TMEM97 (Sigma Intracellular Receptor 2)

**Figure 1:**
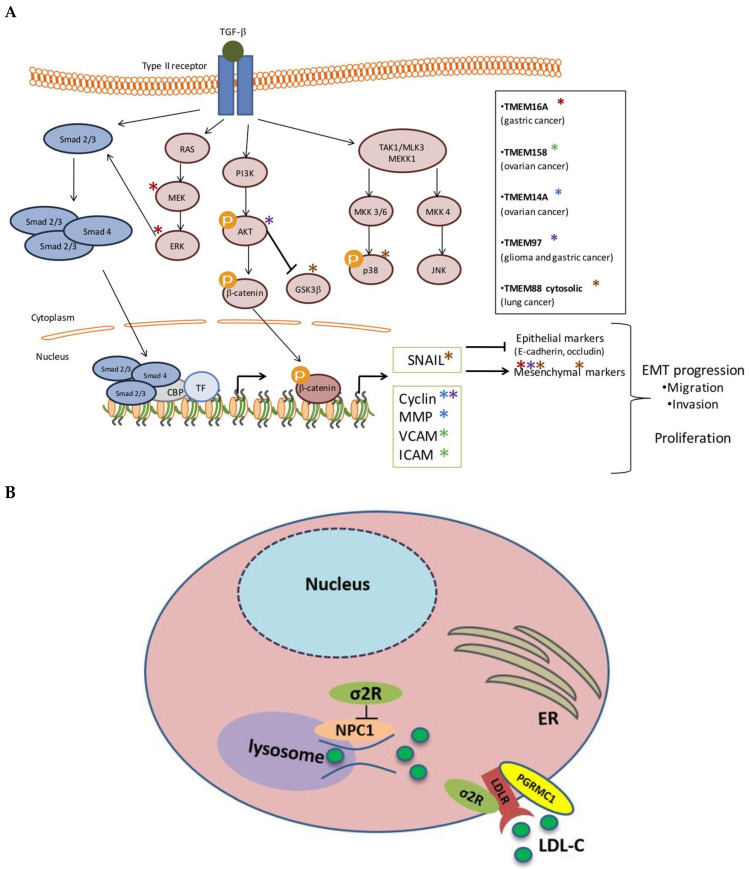
(**A**) TMEM97 promotes cellular proliferation through activation of AKT; the purple star next to AKT indicates the pathway TMEM97 is involved; the other pathways can largely be ignored; EMT, epithelial–mesenchymal transition [67]. (**B**) The internalisation of LDL is mediated by a trimeric complex formed by TMEM97 (σ2R), progesterone receptor membrane component 1 (PGRMC1), and low-density lipoprotein receptor (LDLR). Additionally, TMEM97 is a Niemann-Pick C1 (NPC1) binding protein that regulates cholesterol trafficking out of lysosomes; LDL cholesterol molecules are shown by green spheres. Reproduced with permission CreativeCommons. Available online: http://creativecommons.org/licenses/by/4.0/ (accessed on 23 November 2025) [68].

**Sigma intracellular receptor 2 (TMEM97)** is a transmembrane protein involved in cholesterol homeostasis, cancer development, and neurological functions [63]. Generally, it activates the AKT pathway, leading to increased likelihood of cancer cell proliferation and migration (Figure 1A). In addition, it plays a vital role in regulating cellular cholesterol levels, supporting LDL uptake, and cholesterol transport to the lysosomes (Figure 1B). Indeed, cholesterol-carrying LDL particles bind to transmembrane receptors on the plasma membrane (PM) and are then internalised via receptor-mediated endocytosis; Subsequently, LDL particles are delivered to lysosomes, where the LDL cholesterol is released [63,64]. Specifically, in the liver hepatocyte cell surface, the LDL receptor (LDLR) binds the LDL cholesterol (LDL-C) molecule [65]. This facilitates the formation of a complex that is taken into cells via an endosome. The endosome then transports and delivers LDL-C to lysosomes for degradation under acidic conditions. Finally, the LDLR is recycled back into the cell surface for the process to be repeated. This was identified as one of the four markers that distinguished high-grade HPV+ related lesions from low-grade lesions. TMEM97 has a cell proliferative effect on cancer development and has been associated with dysplasia and cancer pathways [17]. In animal models, TMEM97 has already been identified as a potential biomarker for atherosclerotic plaques [66].

TMEM97 has also been shown to enhance the expression of pro-inflammatory cytokines, including IL-1β, and can therefore potentially worsen inflammatory heart disease [69] as well as negatively affect post-cardiac surgery outcomes. Recent findings also suggest that TMEM97 may play an important role in regulating lipid metabolism following stroke through stress-related endoplasmic reticulum (ER) mechanisms [70]. Specifically, under ER stress conditions, researchers found that TMEM97 expression is reduced, while LDLR and cellular LDL uptake are increased.

#### 2.2.5. IGHA1/2 (Immunoglobulin Heavy Constant Gamma 2)

**Immunoglobulin heavy constant gamma 2 (IGHA1/2)** is a glycoprotein-based antibody secreted by B lymphocytes, which plays an important role in the elimination of foreign antigens as part of the immune response. Three of its glycoforms are significantly downregulated in HPV+ tumours compared to HPV− controls, suggesting that HPV has potential host immune suppression effects [10]. Deficiency in this protein is associated with recurrent bacterial infections [71], which has relevance to cardiovascular disease in terms of i) increased risk of deep sternal wound infection following cardiac surgery (secondary risk in terms of recovery challenge); and ii) increased risk of endocarditis (primary risk).

#### 2.2.6. SERPINA1 (Alpha-1-Antitrypsin)

**Alpha-1-antitrypsin (SERPINA1)** is a serine protease inhibitor that prevents uncontrolled damage to surrounding tissues by neutrophil elastases released during inflammatory responses. It also has moderate binding for thrombin and plasmin. In oropharyngeal cancer, all glycoforms of SERPINA1 are increased in HPV+ cells compared with HPV− and healthy control cells [3]. In relation to the heart, higher levels of Alpha-1-antitrypsin were found during the acute phase of myocardial infarction (MI), with patients who were unable to upregulate this protein having a higher risk of cardiogenic shock and mortality [72]. Whether an increased level of this protein in oropharyngeal or cervical tissues in HPV+ patients would limit the availability of this protein in the heart is largely unknown. One possibility for such a scenario would be that the protein production requires essential amino acids that are limited in supply. In addition, deficiencies in the Alpha-1-antitrypsin protein are associated with decreased heart muscles elasticity, increasing the risk of heart failure [73].

The following proteins were found to be differentially expressed in HPV+ oropharyngeal tumours compared to HPV− tumours: Junctional sarcoplasmic reticulum protein 1, Anoctamin-1, Collagen alpha-1 (XII) chain, Collagen alpha-1 (XVII) chain, Collagen alpha-3 (VI) chain, Fibronectin, Ankycorbin, Periostin, and SPARC [26]. Junctional sarcoplasmic reticulum protein 1 (JSRP1) is upregulated in a mouse model of dilated cardiomyopathy [74]. Anoctamin-1 (ANO1), a calcium-activated chloride channels (CaCCs) protein is increased during ischaemia and is suggested to contribute partly to the development of ischaemia-induced arrhythmias [75]. Anoctamin-1 is also associated with increased cardiac fibrosis in mouse models following myocardial infarction [76], suggesting that its role in arrhythmias is likely linked to its contribution to cardiac fibrosis development [77]. The increased expression of three collagen-related proteins—Collagen alpha-1 (XII) chain, Collagen alpha-1 (XVII) chain, and Collagen alpha-3 (VI) chain—is also associated with tumours from a variety of other cancers in addition to HPV+ cervical cancer, including colorectal (CRC), gastric, breast, bladder, renal, ovarian, pancreatic, and head and neck cancers [78,79,80]. In relation to the heart, accumulation of these proteins is primarily involved in cardiac regeneration [81], cardiogenesis, structural and functional maintenance [82], valve leaflets, and septal development [83].

#### 2.2.7. Nuclear Factor κB Essential Modulator (NEMO), Casein Kinase 1 (CK1), and Beta-Transducin Repeat-Containing Protein (β-TrCP)

One study found that the three proteins **NEMO, CK1, and β-TrCP** were degraded by the HPV E7 oncoprotein in patients with vulvar and cervical cancer [21]. This suggested that the HPV virus was capable of suppressing the host’s antiviral immune response through weakening the NF-κB and Wnt/β-catenin signalling cascades that are mediated by these proteins. This disruption also impairs the expression of innate peptides that support the healthy microbiome conditions in the vagina, contributing to dysbiosis [21].

**NEMO** is the regulatory subunit of the IKK core complex, which phosphorylates NF-κB inhibitors, leading to dissociation of the inhibitor/NF-κB complex and eventual degradation. Knockout of the gene expressing NEMO has previously been shown to result in the development of dilated cardiomyopathy [21] accompanied by increased cardiomyocyte ageing and stress, suggesting a protective, anti-inflammatory role of NEMO in the heart.

#### 2.2.8. Thbs1 (Thrombospondin 1), Kininogen-1 (KNG1), Histidine-Rich Glycoprotein (HRG), Paraoxonase-1 (PON1), and Coagulation Factor X (F10)

Another study applied disease pathways analysis and found that the proteins Thbs1 (or TSP-1), KNG1, Histidine-rich glycoprotein (HRG), and F9, F10, and F12 were upregulated in HPV+ variant P16-induced oropharyngeal tumours compared to non-P16 variant HPV+ tumours [25]. In addition, through disease pathway analysis, it was found that these proteins participated in Cardiovascular System Development and Function (THBS1, KNG1) and Haematological Disease pathways (HRG and F9, F10, and F12) [25]. Using network interaction analysis, PON1 was identified as a differentially expressed across early stage p16+ve OPSCC versus controls, specifically affecting the lipid metabolism pathways [25].

**Thbs1** overexpression is associated with cardiac atrophy during development and with an excess autophagic effect on cardiomyocytes [84].

**KNG1** has been shown to be associated with increased oxidative stress and mitochondrial damage in the heart [85]. In addition, KNG1 is expressed as two proteins, high-molecular-weight kininogen (HK) and low-molecular-weight kininogen (LK), whereby the HK upregulates bradykinin, resulting in increased vascular permeability and inflammation [86]. Furthermore, KNG1 works as part of the kallikrein–kinin system to activate the coagulation cascade [87].

**PON1** is a protein that mediates an enzymatic protection of low-density lipoproteins against oxidative modification and the consequent series of events leading to atheroma formation. Importantly, human PON1 expression prevented the proatherogenic effects of oxidised LDL by lowering its levels in plasma and plaque [88]. This indicates that inhibition of this protein by HPV contributes to atherosclerosis development at least in part through PON1 dysfunction. Immobilisation of PON1 within oxoammonium-functionalised nanogels improved its stability, lipid-protective activities, and antioxidant catalytic activities by releasing the protein only at pH 6 in a glutathione-rich environment [89]. This provides a novel therapeutic delivery strategy that could potentially benefit cardiovascular patients at risk of HPV infection.

**HRG** is a plasma glycoprotein with anticoagulation and antifibrinolytic effects and is capable of binding a variety of ligands, including plasminogen, thrombospondin, heparin, heparan sulphate, haem, and divalent metal ions. Downregulation of HRG has been associated with the development of atherosclerosis and CAD [90].

**F10** and other associated Factor 9 and 12 proteins are involved in clotting and are targeted in anticoagulation management for atrial fibrillation [77].

## 3. Machine Learning and HPV Proteomics

One study combined publicly available protein expression data with transcriptomics data using random forest SVM-RFE and LASSO to derive a set of differentially expressed dysregulated proteins in HPV18-related Neuroendocrine cervical carcinoma [91]. Another study included only demographic characteristics, cervical cancer screening history, HPV infection status, and medical examination results as part of an Xgboost machine learning model to predict cervical cancer prevention (CCP) subgroups to which HPV patients belonged, i.e., (0) healthy, (1) early onset, (2) screening-targeted, (3) late onset, and (4) carcinoma-specific with AUC of 0.995 [92]. However, the limitation is that HPV status was included in the analysis and the study also suggested that the inclusion of proteomics data using a multi-omics approach would have improved the robustness of the model, as well as provided further mechanistic insights.

Using the Cancer Genome Atlas (TCGA) public dataset, another study trained a random survival forest and applied it to an external reverse-phase protein array (RPPA) dataset to predict low-, medium-, or high-risk survival groups for invasive cervical cancer [93]. Analysis of differences in protein signature was then conducted across HPV+ and HPV− groups. Machine and deep learning models using proteomics were mainly used to understand the pathogenesis, diagnose, and predict survival outcomes of cervical cancers, taking into consideration HPV infections [94].

**Figure 2:**
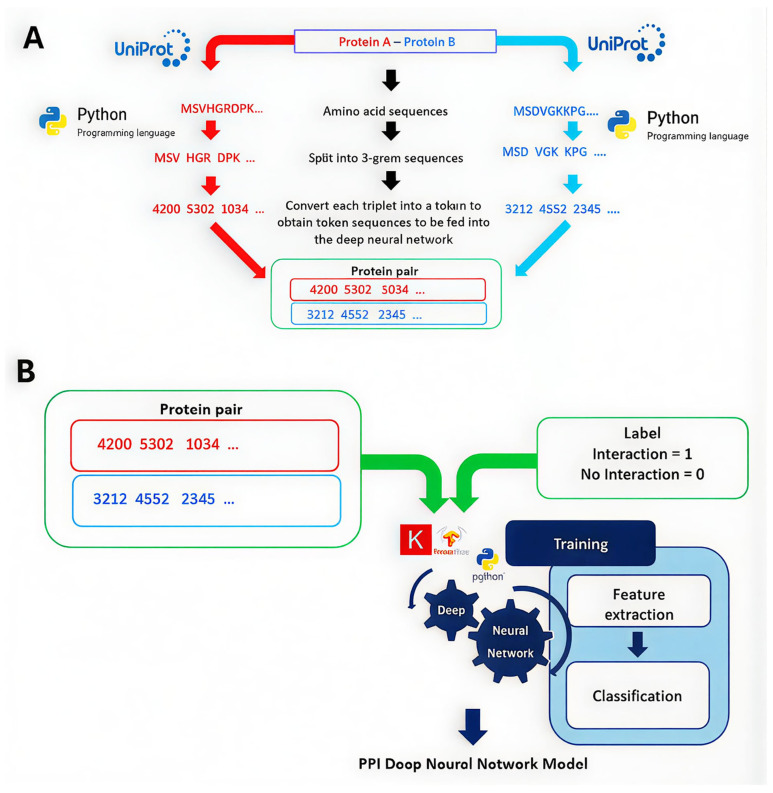
(**A**) Protein sequence pair featurisation process: candidate pairs of amino acid sequences are pre-processed into 3-gram chunks of letters before converting into sets of four integer encoding; (**B**) output of step in (**A**) are fed into a recurrent neural network training process for protein–protein interaction feature extraction and interaction classification; reproduced at highest possible resolution with permission from CreativeCommons. Available online: https://creativecommons.org/licenses/by-nc/4.0/ (accessed on 23 November 2025) [95].

However, a further study focused primarily on protein–protein interactions among HPV+ variants, as well as their interactions with other proteins within the Viruses. STRING public database [95]. The candidate pairs of amino acid sequences were pre-processed into a four-integer encoding before training (Figure 2A). Subsequently, recurrent neural networks were applied in order to obtain a Matthew Correlation Score of 0.79 for the model with key interacting proteins identified for the HPV variants, including AKT, IQGAP1, and MMP16 (Figure 2B).

In relation to the heart, one study used a set of machine learning algorithms, Logistic regression, K-nearest neighbour, Support Vector Machine, and Random Forest to compare the interactions between 3400 host and 3800 pathogen protein targets to determine the targets most associated with cardiovascular disease [96]. The protein targets were identified from DrugBank, and the features (sequence features, post-translational modifications (PTMs), structural, and functional features) were subsequently derived from UniProt for training. Validation was then conducted on MorCVD, the authors’ previously curated host-pathogen protein interaction dataset. Interestingly, this study identified the Major capsid protein L1 (L1) of HPV type 16 as one of the top five pathogen proteins for association with the host cardiovascular disease [96].

Another study used the GEO2R tool (https://www.ncbi.nlm.nih.gov/geo/geo2r/, accessed on 23 November 2025) to identify differentially expressed genes (DEGs) from RNA sequences of patients with dilated cardiomyopathy (DCM) and healthy controls [97]. The DEGs were then screened for disease pathways using the Kyoto Encyclopedia of Genes and Genomes (KEGG) enrichment analysis tool to identify that HPV infection was associated with the presence of DCM DEGs. Machine learning methods (maximum relevance minimum redundancy (mRMR) combined with least absolute shrinkage and selection operator (LASSO) feature selection logistic regression) were then used to reduce the DEGs to a parsimonious set of key genes (*IL1RL1*, *SEZ6L*, *SFRP4*, *COL22A1*, *RNASE2*, *HB*). These key genes were then further screened for previously unknown endocrine-disrupting chemicals that could damage the heart by interacting with them based on an overlapping set from the Comparative Toxicogenomic Database (CTD) and the Endocrine Disruption Exchange website.

## 4. Machine Learning and HPV Transcriptomics

One study developed multiple ML models for use with single-cell RNA (scRNA) sequencing to firstly predict whether the patient had Head and Neck Cancer (HNSCC) or not, and if they did, whether it was due to HPV+ infection or not (i.e., other potential causative factors such as smoking were included in the analysis) [98]. They used the mRMR (Minimum Redundancy and Maximum Relevance) feature selection algorithm to remove features that were highly correlated with each other, retaining only features that were highly correlated with the outcomes. Among the models tested, the three-layered deep learning model with a 50% dropout rate showed the highest performance on both tasks considered [98].

A separate study used a 2D convolutional neural network (CNN) to model whole RNA sequencing data to predict HPV+ and HPV− status in HNSCC patients [99]. The approach was applied to a whole RNA-seq dataset from an in-house HNSCC cohort and an independent HNSCC cohort from The Cancer Genome Atlas (TCGA). The CNN models consisted of three convolutional blocks obtaining a high performance of 0.90 in terms of precision recall AUC. To enhance the interpretability of the models, the model’s saliency (importance) was extracted using the gradient-weighted class activation mapping (grad-CAM) method. These importance values were used to highlight which genes and pathways were most important in a treemap built from RNA expression data [99]. This involved passing the grad-CAM activations mapped onto the 50 MsigDB Hallmark pathway-related genes to build a biological network through the Kamada Kawai layout such that the pathways represent nodes, while the edges are weighted by the proportion of overlapping genes across the nodes.

Whilst not specifically examining HPV prediction, one ML-based study using the SMART-Seq^®^ high throughput (HT) Kit to extract RNA [77] and quantitative reverse transcription PCR (qRT-PCR) to quantify RNA levels identified HPV infection status as a key pathway in patients with breast capsular contracture [100]. The transcriptome was assessed in terms of the competitive endogenous RNA (ceRNA) networks [101], i.e., by examining both mRNA–miRNA interactions and miRNA–lncRNA interactions. Support Vector Machine (SVM) with Recursive Feature Elimination (RFE) was used to determine the set of prognostic genes [100].

Another innovative study applied an unsupervised Louvain clustering method to 11,399 cells from the cervical cancer tissue of a single 53-year-old female patient who had undergone resection [102]. The clustering approach identified 11 epithelial gene clusters applied after reducing 2000 genes to 20 top principal components and visualising them using a UMAP topological model. The clusters were examined in relation to HPV+ status if both *E6* and *E7* viral genes were present or labelled as E6+ and E7+ if only one of these genes was present [102]. The clusters were also compared to normal epithelial gene expression identifying clusters with a high proportion of HPV+ cells as well as the associated genes and enriched pathways.

The Louvain clustering approach was also used in a different study, which combined multiple publicly available microarray RNA expression datasets as a training set, including an RNA sequencing dataset as the test dataset, to predict HPV status, as well as separately clustering the tumour microenvironment in the HPV+ predicted set into five distinct clusters for explainability [103]. The combined datasets were aligned initially using the set of 158 genes described in a previous study but further selected during 5-fold cross-validation to remove genes with low importance, retaining 144 genes based on the model with the best set of hyperparameters in each iteration. The Louvain clustering was separately conducted on the 19 features generated from ssGSEA and PROGENy pathway signature activity score prediction tools. The predicted clusters identified by the Louvain clustering approach were then validated using the K-nearest neighbour algorithm [103].

**Figure 3:**
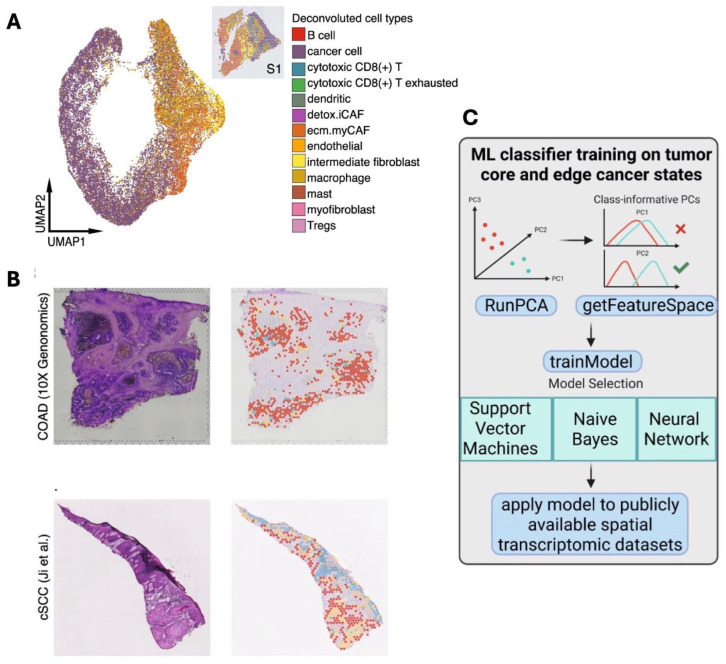
(**A**) CARD-based cell type deconvolution and annotation with uniform manifold approximation and projections (UMAP); single-cell HNSCC transcriptomics data were used to predict the cancer cell types of each cell within a query dataset tissue sample prior to projection. (**B**) Annotation of the four predicted classes on histological images; haematoxylin and eosin (H&E) stained tissue cross-sections (left); tissues labelled with four ML-predicted classes of cancer migration status: tumour core, leading edge, transitory, or other remaining spots; (**C**) The machine learning classification pipeline used. Adapted with permission from CreativeCommons. Available online: http://creativecommons.org/licenses/by/4.0/ (accessed on 23 November 2025) [104].

One study used spatial transcriptomic data analysed from 12 resected HPV-negative oral squamous cell carcinoma (OSCC) tissue samples to predict whether cancer cells belonged to one of four different classes, namely leading edge, transitory, tumour core, or non-cancer, in order to spatially visualise the trajectory of cancer development in OSCC tissues [104]. They applied the CARD deconvolution algorithm using Single-cell HNSCC data as a reference and the spatial transcriptomics data as a query to assign cells with a deconvolution score of >0.99 to different cancers; cells were otherwise classified as non-cancer cell types (Figure 3A). Four predicted classes, based on where the cancer was located, were then used to annotate histological 2D images of the tissues using the ‘SpatialDimPlot’ function of the Seurat R package (Version 4.2.0; Figure 3B). These predictions were generated using three machine learning models, specifically Support Vector Machines with Radial Basis Function Kernel, Model Averaged Neural Network, and Naive Bayes, built using the top 50 class-informative principal components from gene expression data (Figure 3C) [104].

In addition, long-term outcomes following treatment for oropharyngeal carcinoma caused by HPV+ infection have been modelled using random forest machine learning models, whereby data from three modalities (i.e., clinical data, radiological data, and histological data) were trained following initial univariate feature filtering [105]. Evaluation using 5-split stratified cross-validation predicted 5-year risk of relapse-free survival with satisfactory performance of F1-score 81.08% and AUC 0.89.

A pan-cancer ResNet-based deep learning model has also been built to predict cancer vs. no cancer status using histological slide images from six cancer types, including HNSCC [106]. The features extracted by the deep learning model were then combined with both proteomic and transcriptomic data through a sparse multi-canonical correlation analysis (Multi-CCA) model. This Multi-CCA model enabled the provision of an additional explanation of genes and proteins associated with the largest and least canonical weights based on corresponding pathways from gene ontology (GO) term enrichment [106]. The idea was to understand which genes or proteins were associated strongly with particular histology slides in cancer tissues, with pathologists helping to annotate the slides to understand how these are expressed under different scenarios.

Furthermore, a study used a meta-learning frame called MAML with a four-layered deep neural network to integrate transcriptomics data with proteomics and clinical data for the prediction of the presence of three different cancer types, including HNSCC [107]. To combine the deep neural network with cox proportional hazard, the cox hazard loss function was applied. Furthermore, a backpropagation-based deep model explanation tool called DeepLIFT was applied to understand the importance of variables [107]. Finally, a study directly predicted the HPV+ HNSCC status using NF-κB activity-related genes using a nearest centroid classifier [108].

## 5. Clinical Diagnostics and Therapeutics

In terms of practical application within the diagnostic laboratory in the United Kingdom (UK), PCR screening of HPV RNA is currently used due to the reduced false positive rate of RNA compared to DNA. However, the limitation is that unlike mass spectrometry, there are a limited number of HPV variants that can be detected using the PCR approach, making it difficult to adjust to the emergence of new high-risk HPV variants. On the other hand, mass spectrometry has sample preparation-related noise that is typically more adapted for bacterial data, which involves culturing prior to analysis. The greater suitability of HPV for RNA amplification will likely continue to limit the use of mass spectrometry for clinical application. Nonetheless, PCR-coupled mass spectrometry may be a way to overcome such limitations [109].

Currently, the majority of HPV biomarker-related studies are experimental rather than clinical. In addition, it should be noted that one of the major limitations of UK HPV laboratory diagnosis is that only the HPV+ or HPV− status is reported, with no information of genetic sequence, specific variant types, or molecular information provided that could support monitoring and understanding of the relationship between viral strains and patient outcomes for either quality management or research purposes.

Recent research has examined the interactions between molecular markers across the heart and the gut, showing that following myocardial infarction, the gut lining becomes more permeable [110]. This makes these patients more susceptible to heart infections by bacteria such as *E. coli*, and probiotic strains of this species were engineered to counter infections by providing cardio-protective benefits. However, no infections in the context of HPV were considered.

**Figure 4:**
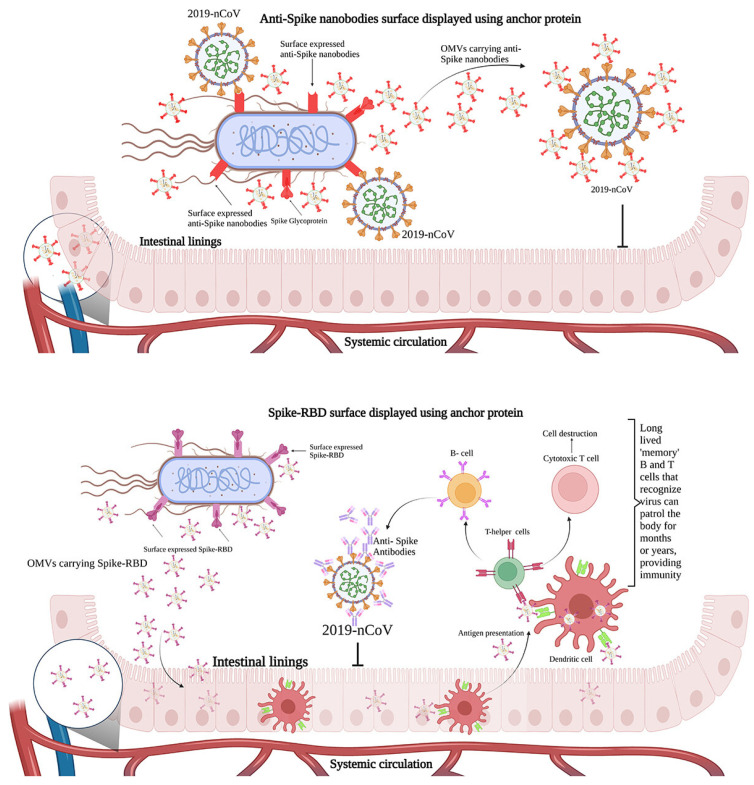
Engineered *Escherichia coli Nissle* 1917 (EcN) functions as a platform for SARS-CoV-2 vaccination and oral antiviral therapy. Top panel: modified EcNs neutralise the virus in the stomach and may even penetrate the systemic circulation by releasing bacterial outer membrane vesicles (OMVs) bearing anti-spike nanobodies on their surface. Bottom panel: spike protein’s receptor-binding domain (Spike-RBD) is expressed by modified EcN, which stimulates dendritic cells to deliver antigens, triggering T cells to produce antibodies and B cells to retain immunological memory. Mucosal and systemic immunity against SARS-CoV-2 is provided by this method; reproduced with permission from CreativeCommons. Available online: http://creativecommons.org/licenses/by/4.0/ (accessed on 23 November 2025) [111].

Vaccination strategies by another study have also focused on the use of engineered bacteria, such as *Escherichia coli* Nissle 1917 (EcN), that can be easily administered orally without injection [111]. The approach enables viral spike-binding nanobodies to be secreted directly from the bacteria and simultaneously presenting the viral antigen on the bacterial surface to trigger immune generation of antibodies to neutralise the virus both in the gut and systemically (Figure 4). A similar strategy could also be developed for tackling the HPV infection.

However, because HPV testing is primarily performed on women in conjunction with cervical cancer screening, there are no NHS-reported data available for male patients. Hence, additional studies would be beneficial in the male population to conduct HPV vaccination trials to understand the effect this has on cardiovascular diseases [8,14].

In the UK diagnostic laboratory, HPV+ detection through PCR is typically followed by a biomedical scientist’s manual check of cervical smear slides for morphological changes in cells to determine the stage of HPV infection, including, for example, no change, pre-cancer, or cancerous. The initial primary screener determines whether there is a cell change. If a change is detected, this is then checked by a secondary screener, before being approved by a senior biomedical scientist and a consultant who adjudicates any conflicting opinions. Whilst artificial intelligence screening could potentially replace the role of the primary screener, due to potentially different staining procedures used by AI algorithms, the checking time for the secondary screener could take longer, potentially reducing any advantage in terms of speed gained by the AI system. In addition, the worry and potential for the impact of such changes on the workforce in terms of modifying opportunities and changes in the roles of existing biomedical scientists could all hinder clinical AI adoption. Hence, any future technology implementations need to minimise discrepancies with existing sample preparation processes to minimise the need to re-train scientists to these processes, as well as take into account anthropological factors to prevent the likelihood of costly research and development failures for the technology providers.

## 6. Conclusions and Future Perspective

Although there is some information regarding high-level associations between HPV infection and CVD in the literature, there is currently a lack of understanding of the mechanisms behind these interactions. This review aimed to address this gap by further describing the proteomic and transcriptomic markers shared across these two disparate conditions. In addition to known existing relationships between HPV, atherosclerosis, and CAD, this review additionally identified dilated cardiomyopathy as a key cardiovascular disease modified by HPV infections. Importantly, it suggests that DCM may be mediated by HPV-induced degradation of the NEMO protein, which has previously been shown to lead to dilated cardiomyopathy, accompanied by increased cardiomyocyte ageing and stress.

In addition, this review provides a more comprehensive understanding of cholesterol-modifying mechanisms behind HPV’s influence on CVD through (i) the binding of isoform Hp2-2 to apolipoproteins to disrupt the reverse cholesterol transport process; (ii) abnormal levels of TMEM97, which further disrupt low-density lipoprotein (LDL) storage and metabolism; (iii) KNG1, whose HK protein results in the permeability and inflammation of blood vasculature; (iv) downregulation of anticoagulation and antifibrinolytic proteins such as HRG; (v) upregulation of coagulation factors such as F9, F10, and F12 increase thrombotic effects; (vi) the cardiomyocyte senescence effect of p16 combined with the excess autophagic effect of Thbs1 overexpression on cardiomyocytes results in increased availability of blood cell lysis residuals from autophagy-induced cell death that may further enhance atherogenesis together with the effect of increased platelet activation; and (vii) the viral downregulation of PON1 protein degrades the atheroprotective mechanism of the host [112]. Taken together, these suggest that the increased permeability of blood vessels combined with the cholesterol/lipid modification effects of HPV could potentially lead to increased absorption of lipids, especially LDL, and autophagic-induced blood cell lysis components into the endothelial surface of blood vessels, fuelled by increased thrombotic driving effects of coagulation factors, increasing the risk of atherosclerosis and CAD. The inflammatory effects and tendency for clotting and atherogenesis are further enhanced as a result of the downregulation of important antioxidant and protective mechanism-inducing proteins such as NEMO. However, further in-vitro and in-vivo studies may be required to further explore these findings, as well as to identify other cardiovascular effects of proteins differentially expressed in HPV infection-related tumours across a range of tissues.

Whilst limited literature exists on the use of machine learning for the analysis of HPV proteomics, especially in relation to cardiovascular disease, future studies should further the development and application of ML to better utilise the multimodality data sources facilitating the linkage across heterogeneous proteomics, electronic health records (EHR), transcriptomics, digital pathology, and imaging and genomics datasets. It is envisaged that proteomic atlases including more comprehensive metadata such as sequence features, post-translational modifications (PTMs), and structural and functional features could facilitate the development of such machine learning models alongside more HPV variant-specific features such as RNA sequences. Furthermore, the integration of the biomarkers compiled here should also aim to support the development of models that are more tailored to the understanding, diagnosis, and management of HPV infections, as well as the potentially associated cardiovascular complications. One area that has particularly troubled cardiac surgeons is the lack of understanding regarding why female patients tend to have a higher EuroSCORE II risk [113]. Further work alongside this current work may help to disentangle the complexities of such idiopathic (i.e., unknown) components of the risk score.

Furthermore, whether HPV vaccination could have a protective effect on the risk of cardiovascular disease will require further research. Recent research has examined the interactions between molecular markers across the heart and the gut, showing that following myocardial infarction, the gut lining becomes more permeable [110].

## Figures and Tables

**Table 1:** Known/suspected biomarkers associated with HPV pathogenesis.

Category	Tissue Type	Biomarkers	References
**Proteomics**	Vulvar and cervical	ASF1B, **CDC20**, UBE2C and ATAD2; TNFRSF12A/Fn14, OLFM1 and **ID1**	[16]
		EIF1; BLOC1S5; LIMCH1; SGTA; ERH; IGKV2-30; TMEM97; DNAJA4	[17]
		LDHA	[18]
		Involucrin; IL-18; Probable protein E4; Major capsid protein **L1**; Early protein E4; Minor capsid protein L2; E4 protein; L2 protein	[19]
		Collagen type I alpha 2 and alpha 1 (COL1A1 and 2); periostin osteoblast specific factor (POSTN) and fibrillin 1 (FBN1)	[20]
		NEMO, CK1 and β-TrCP	[21]
	Head and neck squamous cell	ULK1	[22]
		NRF2, p16, TP73	[23]
	Oral and oropharyngeal	CPPED1, GPRC5A, and TAGLN; CPPED1, OAS2, OAS3, FN1, SAMHD1, and ISG15; KYNU, LCP1, UCHL1, and GAGE12H	[24]
		A1BG, AHSG, AMBP, APOA4, APOC1, APOC2, APOC4; APOD, APOF, APOH, APOL1, APOM; ORM2, PON1; SAA1, SAA2, SAA2-SAA4; SERPINF1, SERPINF2, SOD3; TTR, UBA3	[25]
		HP, IGHA1/2, SERPINA1	
		THBS1, KNG1, HRG, F9,F10,F12	[25]
		JSRP1, ANO1, Collagen alpha-1 (XII) chain, Collagen alpha-1 (XVII) chain, Collagen alpha-3 (VI) chain, Fibronectin, Ankycorbin, Periostin, SPARC	[26]
**Transcriptomics**	Cervical cell	*E6/E7*	[27]
		*HPV16-miR-H1-1*, *HPV16-miR-H2-1*	[28]
		*CCNB2, PRC1, SYCP2, **CDC20**, NUSAP1, CDKN3*	[29]
		***L1***	[30]
		***Id-1***	[31]
		*miR-146a-5p*	[32]
		*PA2G4, ATL3*	[33]
		*S100P; KRT17; PDE3A; TM4SF1; TLR4; AQP3*	[34]
		*TMEM45A, SERPINB5 and p16INK4a*	[35]
	Oropharyngeal	*miR-9*	[36,37]
		*E6/E7*	[38]
		*Stress keratin 17 (K17)*	[39]
	Keratinocytes	*NFX1-123*	[40]
		*miR-9-5p, miR-363-3p*	[37]
		*ΔNp73α*	[41]

## Data Availability

No new data were created or analysed in this study. Data sharing is not applicable to this article.
